# Association between mild cognitive impairment and trajectory-based spatial parameters during timed up and go test using a laser range sensor

**DOI:** 10.1186/s12984-017-0289-z

**Published:** 2017-08-08

**Authors:** Shu Nishiguchi, Ayanori Yorozu, Daiki Adachi, Masaki Takahashi, Tomoki Aoyama

**Affiliations:** 1 Department of Physical Therapy, School of Health Sciences, Tokyo University of Technology, Tokyo, Japan; 2 Graduate School of Science and Technology, Keio University, Yokohama, Japan; 3 Department of Physical Therapy, Human Health Sciences, Graduate School of Medicine, Kyoto University, Kyoto, Japan; 4 Department of System Design Engineering, Faculty of Science and Technology, Keio University, 3-14-1 Hiyoshi, Kohoku-ku Yokohama, 223-8522 Japan

**Keywords:** Timed up and go test, Mild cognitive impairment, Older adults, Laser range sensor, Trajectory-based spatial parameters

## Abstract

**Background:**

The Timed Up and Go (TUG) test may be a useful tool to detect not only mobility impairment but also possible cognitive impairment. In this cross-sectional study, we used the TUG test to investigate the associations between trajectory-based spatial parameters measured by laser range sensor (LRS) and cognitive impairment in community-dwelling older adults.

**Methods:**

The participants were 63 community-dwelling older adults (mean age, 73.0 ± 6.3 years). The trajectory-based spatial parameters during the TUG test were measured using an LRS. In each forward and backward phase, we calculated the minimum distance from the marker, the maximum distance from the *x*-axis (center line), the length of the trajectories, and the area of region surrounded by the trajectory of the center of gravity and the *x-*axis (center line). We measured mild cognitive impairment using the Mini-Mental State Examination score (26/27 was the cut-off score for defining mild cognitive impairment).

**Results:**

Compared with participants with normal cognitive function, those with mild cognitive impairment exhibited the following trajectory-based spatial parameters: short minimum distance from the marker (*p* = 0.044), narrow area of center of gravity in the forward phase (*p* = 0.012), and a large forward/whole phase ratio of the area of the center of gravity (*p* = 0.026) during the TUG test. In multivariate logistic regression analyses, a short minimum distance from the marker (odds ratio [OR]: 0.82, 95% confidence interval [CI]: 0.69–0.98), narrow area of the center of gravity in the forward phase (OR: 0.01, 95% CI: 0.00–0.36), and large forward/whole phase ratio of the area of the center of gravity (OR: 0.94, 95% CI: 0.88–0.99) were independently associated with mild cognitive impairment.

**Conclusions:**

In conclusion, our results indicate that some of the trajectory-based spatial parameters measured by LRS during the TUG test were independently associated with cognitive impairment in older adults. In particular, older adults with cognitive impairment exhibit shorter minimum distances from the marker and asymmetrical trajectories during the TUG test.

## Background

Dementia is affecting 5–8% of the population aged 65 and older [1], and up to 30% of people aged 85 and older [2]. Dementia drastically impacts daily life, and its prevalence is increasing overall; approximately 48% of people with Alzheimer’s disease (AD) live in Asia, and this percentage is projected to increase to 59% by 2050 [3]. Although dementia, including AD, is associated with mortality [4], evidential treatment strategies in daily living have not been detected. Thus, effective means of detecting and preventing dementia which can use in daily activity onset must be developed (e.g. medication, exercise, food therapy).

The Timed Up and Go (TUG) test is a simple, quick, and well-established test of lower extremity function and mobility [5]. It requires an individual to stand up from a seated position with his/her arms on the thighs, walk 3 m, turn around, walk back to the chair, and sit down. TUG requires no special equipment or training and is easily included as part of a routine examination of physical function for older adults. Recent research has shown that the TUG test is associated with cognitive function, quantitatively [6–9]. Compared with cognitively healthy older adults, older adults with AD showed poor TUG performance [10]. A longitudinal study indicated that a slow TUG time predicted cognitive decline after 3 years [6]. Furthermore, executive function [7–9], memory, and processing speed [9], as well as global cognitive function [6], were considered to be associated with TUG performance. Thus, the TUG test may be a useful tool to detect not only mobility impairment but also possible cognitive impairment.

Some research groups have performed a quantitative analysis of movement during the TUG test for assessment of cognitive decline, using inertial sensor data, in which studies participants were older adults [11] and patients with Parkinson’s Disease [12]. Similar to that previous study, in which various spatial and temporal parameters such as stride length (spatial parameter) and cadence (temporal parameter) during the TUG test were measured, we have analyzed these parameters during the TUG test, using a laser range sensor (LRS) [13]. An LRS can assess temporal and spatial gait parameters by measuring the distance and angle of the legs, and tracking the trajectory of both legs using an infrared laser sensor. The previous studies investigated that the spatial and temporal parameters during TUG such as stride length, cadence, angular range, and angular velocity were associated with cognitive functions [11, 12]. On the other hand, it has been reported that the gait symmetry and regularity of parameters such as stride length for left and right legs measured by a tri-axis accelerometer was associated with cognitive function [14]. In addition to these, in this study, to examine the relationship between the cognitive function and the walking strategy during the TUG test, that is, how the participants approach the marker and how far they keep the distance from it while turning. Therefore, to assess the walking strategy during the TUG test, it is required for the system to measure the trajectory-based spatial parameters such as the minimum distance between the leg trajectory and the marker, the length of the trajectories, and the area of region surrounded by the trajectory of the center of gravity and the center line (*x*-axis) in the TUG field coordinate system. To measure these trajectory-based spatial parameters in the TUG field coordinate system, we have used the LRS leg tracking system which is able to align the coordinate system. In the LRS system, the LRS position is able to be calibrated using two poles in the TUG field coordinate system easily. Therefore, the system is able to directory measures trajectory-based spatial parameters in the TUG field. In addition, we used the LRS system, which is a non-contact measurement system and realizes smooth measurement because it is necessary to assess many participants in a short time in actual community health centers. The purpose of this cross-sectional study was to investigate whether the measurements of trajectory-based spatial parameters obtained by LRS during the TUG test were in fact associated with cognitive impairment in community-dwelling older adults.

## Methods

### Subjects

This cross-sectional observational study was carried out in the Kyoto prefecture in Japan in September 2014. Volunteers were recruited by advertisements in the local press. We obtained informed consent from each individual included in the study. Eligibility was determined by interview, and the inclusion criteria were as follows: age ≥ 60 years, community resident, ability to ambulate independently with or without an assistive device (cane or walker), and ability to hear and understand an explanation of the TUG test protocol. Since the following diseases are known to affect the results of the TUG test and other motor function tests, we excluded individuals with severe cognitive or neurological disorders, such as Parkinson’s disease and stroke. As a result, a total of 63 older adults (mean age, 73.0 ± 6.3 years) were recruited in this study. The demographic data of the participants are presented in Table 1. This study was conducted in accordance with the human rights guidelines of the Declaration of Helsinki, and the study protocol was reviewed and approved by the Ethics Committee of the Kyoto University Graduate School of Medicine.

**Table 1:** Baseline characteristics of the participants with and without mild cognitive impairment

	All (*n* = 63)		
	Normal group (*n* = 38)	Mild cognitive impairment group (*n* = 25)	*p*-value
Age, (y)	73.2 ± 6.4	72.8 ± 6.2	0.827
Female, n (%)	33 (86.8%)	21 (84.0%)	0.752
BMI, (kg/m^2^)	21.9 ± 3.3	21.7 ± 3.2	0.798
Education (y)	11.8 ± 2.3	11.8 ± 3.2	0.992
Walking speed (m/s)	1.39 ± 0.19	1.42 ± 0.24	0.609
Hand grip strength (kg)	25.3 ± 6.4	24.3 ± 7.6	0.596
TUG time (s)	6.78 ± 1.1	7.12 ± 1.87	0.362
MMSE scores	28.9 ± 1.2	24.7 ± 1.7	< 0.001^**^

Mild cognitive impairment was defined as the cut-off of the MMSE score (26/27)

*BMI* body mass index, *TUG* Timed Up and Go test, *MMSE* Mini-Mental State Examination

*p < 0.05, **p < 0.01

### Demographic data

Data on age, sex, body mass index (BMI), years of education, walking speed, and handgrip strength were obtained. All data were collected in a single session. Information on age, sex, and years of education was obtained directly from the participants, and BMI was calculated from measured height and weight, using standardized height and weight scales. For walking speed measurements, participants were asked to walk 15 m at a speed that was comfortable for them, and the time taken to walk 10 m of that distance was measured using a stopwatch (2.5 m at the start and end were allocated to acceleration and deceleration). Handgrip strength was measured with a standard handgrip dynamometer (Smedley Dynamometer; TTM, Tokyo, Japan). The participants were asked to stand up and hold the dynamometer with their arms parallel to but not touching their bodies. Handgrip strength was measured once for each hand, and the average value (kg) from both hands was used.

### TUG test protocol

The TUG test protocol was based on a previous study [5] and performed as follows: when the participant hears a buzzer from a personal computer, the participant stands up from a standard chair without armrests with a seat height of 40 cm walks 3 m as quickly as possible, turns at a marker, returns to the chair, and sits down. The TUG time was defined as the time from the moment the buzzer sounded to the moment the subject sat back down on the chair, detected automatically using a piezoresistive pressure sensor located under the seat.

### TUG data collection and analysis with the LRS

**Fig. 1:**
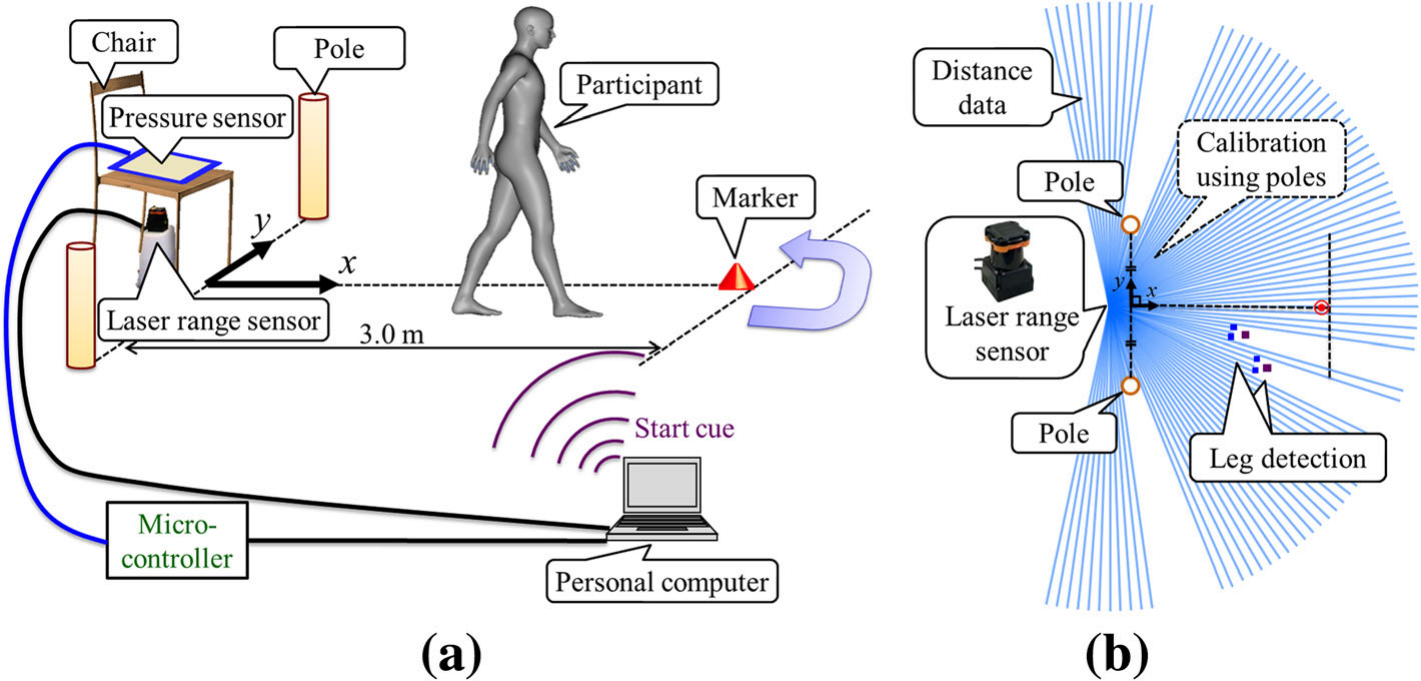
Overview of the Timed Up and Go (TUG) measurement system by use of a laser range sensor (LRS) in the TUG field coordinate system. **a** System configuration, (**b**) Top view of LRS distance data and leg detection

**Fig. 2:**
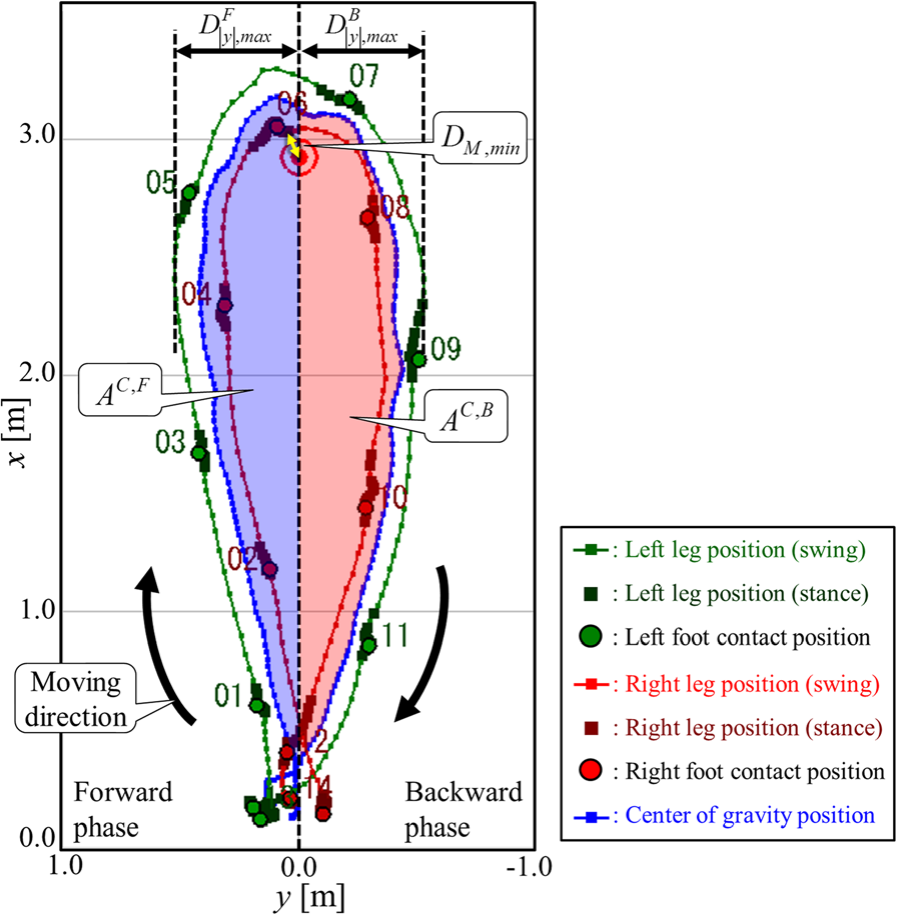
TUG measurement results and trajectory-based spatial parameters

Spatial and temporal raw data obtained during the TUG test were collected using our own system composed of an LRS (UTM-30LX, Hokuyo Automatic Co., Ltd., Osaka, Japan), a piezoresistive pressure sensor (SEN-08713, SparkFun Electronics, Inc., Colorado, United States), a microcontroller (mbed NXP LPC1768, NXP Semiconductors N.V., Einthoven, Netherlands), two calibration poles, and a personal computer. Fig. 1 shows the overview of the TUG measurement system. The LRS was installed at shin height (27 cm above the ground in our system) and it captured two-dimensional distance data by scanning a single infrared laser beam in a horizontal plane at a frequency of 40 Hz as shown in Fig. 1 (b). As shown in Fig. 1 (b), the system calibrated the LRS position in the TUG field coordinate system using two calibration poles which were symmetrically placed at the left and right sides of the TUG field [13]. The system measured the position and velocity of both legs in the TUG field coordinate system based on the characteristic leg patterns determined by the LRS scanned data shown in Fig. 1 (b). We used an algorithm for leg detection and tracking that was proposed in our previous studies [13, 15]. The times for movement initiation and step completion were obtained from the leg position and step velocity data. From validation compared to a force plate [16], foot-off time was defined as the time that leg movement velocity started to increase, while foot-contact time was defined as the time that leg movement velocity was less than the threshold (0.47 m/s) [13]. Temporal data as to the initiation and end of the TUG test protocol were obtained from pressure sensor data (40 Hz). Stand up and sit down times were detected from the change in voltage of a pressure sensor. A computer extracted the spatial and temporal parameters using a program written in Microsoft Visual C+ (Microsoft Japan Co., Ltd., Tokyo, Japan). Details of the data analysis algorithms used in this system were described in our previous study [15]. Fig. 2 shows an example of the measurement results obtained during the TUG test. We have previously tested the accuracy in leg trajectory calculation of the method based on an LRS. This was performed by comparing estimates with a 3D motion tracking system in 10 healthy young adults. The root mean squared error of the *x* and *y* coordinates of the leg trajectories were respectively 0.047 m and 0.028 m [13].

In this study, to examine the relationship between walking strategy during the TUG test and cognitive impairment, the following trajectory-based spatial parameters in each forward and backward phase in the TUG field were also measured: the minimum distance from the marker, the maximum distance from the *x*-axis, the length of the trajectories, and the area of region surrounded by the trajectory of the center of gravity and the *x*-axis (We call it area of the center of gravity). The minimum distance from the marker *D*_*M*  , min_ was the closest distance to the marker of both legs. It was calculated as the minimum distance between the center of the marker and both leg trajectories. In the case of Fig. 2, it was the minimum distance between the marker and right leg trajectory. As shown in Fig. 2, the maximum distances (the absolute values of the *y* coordinate) of both leg trajectories from the *x* axis in the forward and backward phases, ${D}_{\left|y\right|,\mathit{\max}}^{phase}$, were calculated, respectively. The superscript *phase* was *F* or *B*, where *F* and *B* indicate the forward and backward phase during the TUG test, respectively. The trajectory lengths of the center of gravity in each phase were calculated as follows:

1 $$ {L}^{C, phase\kern0.5em =\kern0.5em }\sum \limits_{k={K}_{init}^{phase}}^{K_{end}^{phase}-1}{\left\Vert \boldsymbol{P}\right.}_{k+1}^C-\left.{\boldsymbol{P}}_k^C\right\Vert =\sum \limits_{k={K}_{init}^{phase}}^{K_{end}^{phase}-1}\sqrt{{\left({x}_{k+1}^C-{x}_k^C\right)}^2+{\left({y}_{k+1}^C-{y}_k^C\right)}^2} $$

**Fig. 3:**
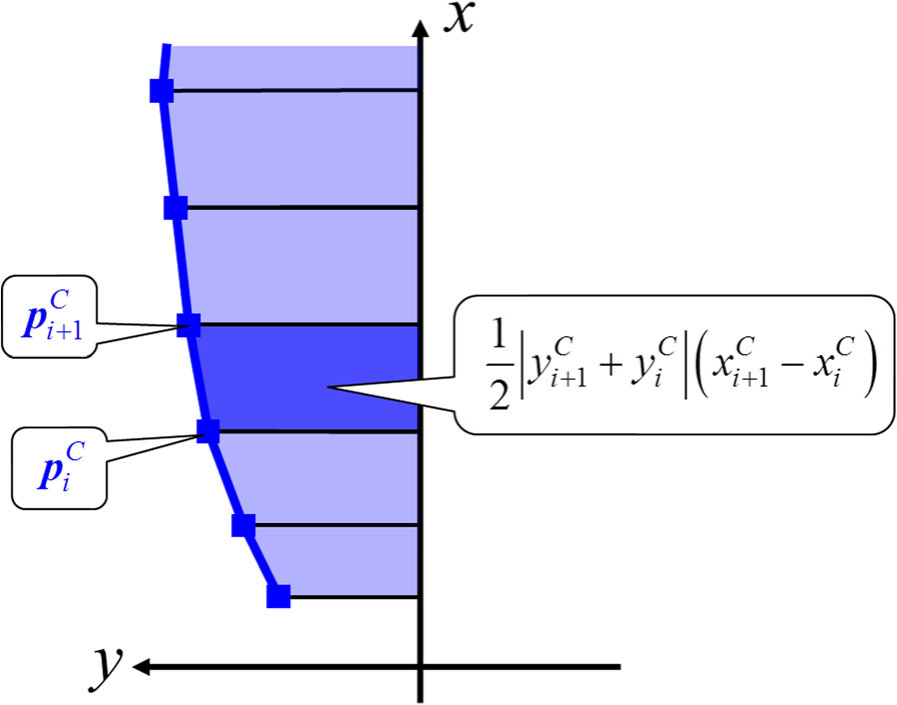
Calculation of the area of the center of gravity from the *x*-axis

Where ${\boldsymbol{P}}_k^C={\left[{x}_k^C\kern1em {y}_k^C\right]}^T$ was the position of the center of gravity at time step *k*, and ${K}_{init}^{phase}$ and ${K}_{end}^{phase}$ were the initial and end time steps in each phase, respectively. As shown in Fig. 3, the areas of region surrounded by the trajectory of the center of gravity and the *x* axis in each phase were calculated as follows:

2 $$ {A}^{C, phase}=\frac{1}{2}\sum \limits_{i={I}_{init}^{phase}}^{I_{end}^{phase}-1}\left|{y}_{i+1}^C+{y}_i^C\right|\left({x}_{i+1}^C-{x}_i^C\right) $$

Where ${\left[{x}_i^C\kern0.5em {y}_i^C\right]}^T$ indicates the *i*-th position of the center of gravity in each phase. ${I}_{init}^{phase}$ and ${I}_{end}^{phase}$ were the initial and end steps in each phase for calculating the area.

### Cognitive function

The participants’ cognitive function was assessed using the Mini-Mental State Examination (MMSE) [17], which is a standard test to assess mental status in cognitive aging research. The MMSE is a short screening test that consists of the following five areas of cognitive impairment detection: orientation, registration, attention and calculation, memory, and language. The maximum possible scores on each subsection are 10, 3, 5, 3, and 9, respectively. In total, the test contains 11 questions, with the total MMSE score ranging from 0 to 30, with a higher score indicating better cognitive performance. We used 26/27 (≤26: mild cognitive impairment, >26: normal). as the cut-off score for defining mild cognitive impairment. According to a previous study, this cut-off has been shown to exhibit maximum diagnostic accuracy for highly educated individuals [18]. In a study by Yukutake et al., this cut-off was used because the participants were living independently in a community and were highly educated [19]. As our participants were also community-dwellers and the distribution of the educational history in our study was similar to that of the study by Yukutake et al., we used this cut-off for detecting cognitive impairment.

### Statistical analysis

Prior to the analysis, we classified the participants into two groups (normal group and mild cognitive impairment group), based on the cut-off score of the MMSE (26/27). The differences in the demographic variables between these two groups were statistically analyzed using the unpaired t-test for continuous variables and the χ^2^ test for categorical variables.

The minimum distance from the marker, the maximum distance from the *x*-axis in each phase, the length of the trajectories in each phase, the area of the center of gravity in each phase, and the forward/whole phase ratio of each parameter (the maximum distance from the *x*-axis, the length of trajectories, and the area of the center of gravity) between the two groups were analyzed using the unpaired t-test.

In addition, multivariate logistic regression analyses adjusted for age, sex, BMI, and TUG time were carried out to determine whether the trajectory-based spatial parameters were associated with mild cognitive impairment. In this analysis, the presence or absence of mild cognitive impairment was used as the dependent variable (normal = 0, mild cognitive impairment = 1), whereas the parameters described above, which showed significant differences in the unpaired t-test, were used as independent variables.

Data were quantified using odds ratios (ORs) with 95% confidence intervals (CIs). The threshold for statistical significance was set at *p* < 0.05. All statistical analyses were performed using SPSS Statistics for Mac OS, version 22.0 (IBM Corp, Armonk, NY, USA).

## Results

Demographic data concerning the participants in both groups are shown in Table 1. There were 38 participants (60.3%) in the normal group and 25 participants (39.7%) in the mild cognitive impairment group. While there were no significant differences in age, sex, BMI, years of education, walking speed, handgrip strength, and TUG time, a significant difference in MMSE score (normal group: 28.9 ± 1.2, mild cognitive impairment group: 24.7 ± 1.7, *p* < 0.001) was observed.

Table 2 shows the group differences of the trajectory-based spatial parameters during the TUG test. The minimum distance from the marker was significantly different between the two groups (normal group: 15.3 ± 3.9 cm, mild cognitive impairment group: 13.2 ± 3.9 cm, *p* = 0.044). The area of the center of gravity in the forward phase (normal group: 0.72 ± 0.17 m^2^, mild cognitive impairment group: 0.61 ± 0.16 m^2^, *p* = 0.012) and the forward/whole phase ratio of the area of the center of gravity (normal group: 49.3 ± 10.8%, mild cognitive impairment group: 42.9 ± 10.7%, *p* = 0.026) were significantly different between the two groups. There were no significant differences in either the minimum distance from the marker and the maximum distance from the *x*-axis.

**Table 2:** Group comparisons of the trajectory-based spatial parameters during the TUG test

	All (*n* = 63)		
	Normal group (*n* = 38)	Mild cognitive impairment group (*n* = 25)	*p*-value
The minimum distance from the marker *D*_*M* , min_ (cm)	15.3 ± 3.9	13.2 ± 3.9	0.044^*^
The maximum distance from *y-*axis in forward phase ${D}_{\left|y\right|,\mathit{\max}}^F$ (m)	0.47 ± 0.07	0.45 ± 0.06	0.352
The length of trajectories in forward phase *L* ^*C* , *F*^ (m)	3.45 ± 0.10	3.46 ± 0.13	0.840
The area of the center of gravity in forward phase *A* ^*C* , *F*^ (m^2^)	0.72 ± 0.17	0.61 ± 0.16	0.012^*^
The maximum distance from *x*-axis in backward phase ${D}_{\left|y\right|,\mathit{\max}}^B$ (m)	0.47 ± 0.08	0.46 ± 0.11	0.483
The length of trajectories in backward phase *L* ^*C* , *B*^ (m)	3.43 ± 0.14	3.40 ± 0.17	0.408
The area of the center of gravity in backward phase *A* ^*C* , *B*^ (m^2^)	0.78 ± 0.21	0.75 ± 0.25	0.573
The forward/whole phase ratio of maximum distance from *x*-axis (%)	50.0 ± 5.9	50.4 ± 6.5	0.785
The forward / whole phase ratio of length of trajectories (%)	50.1 ± 1.1	50.4 ± 1.5	0.410
The forward / whole phase ratio of area of the center of gravity (%)	49.3 ± 10.8	42.9 ± 10.7	0.026^*^

Mild cognitive impairment was defined as the cut-off of the MMSE score (26/27)

*p < 0.05, **p < 0.01

**Fig. 4:**
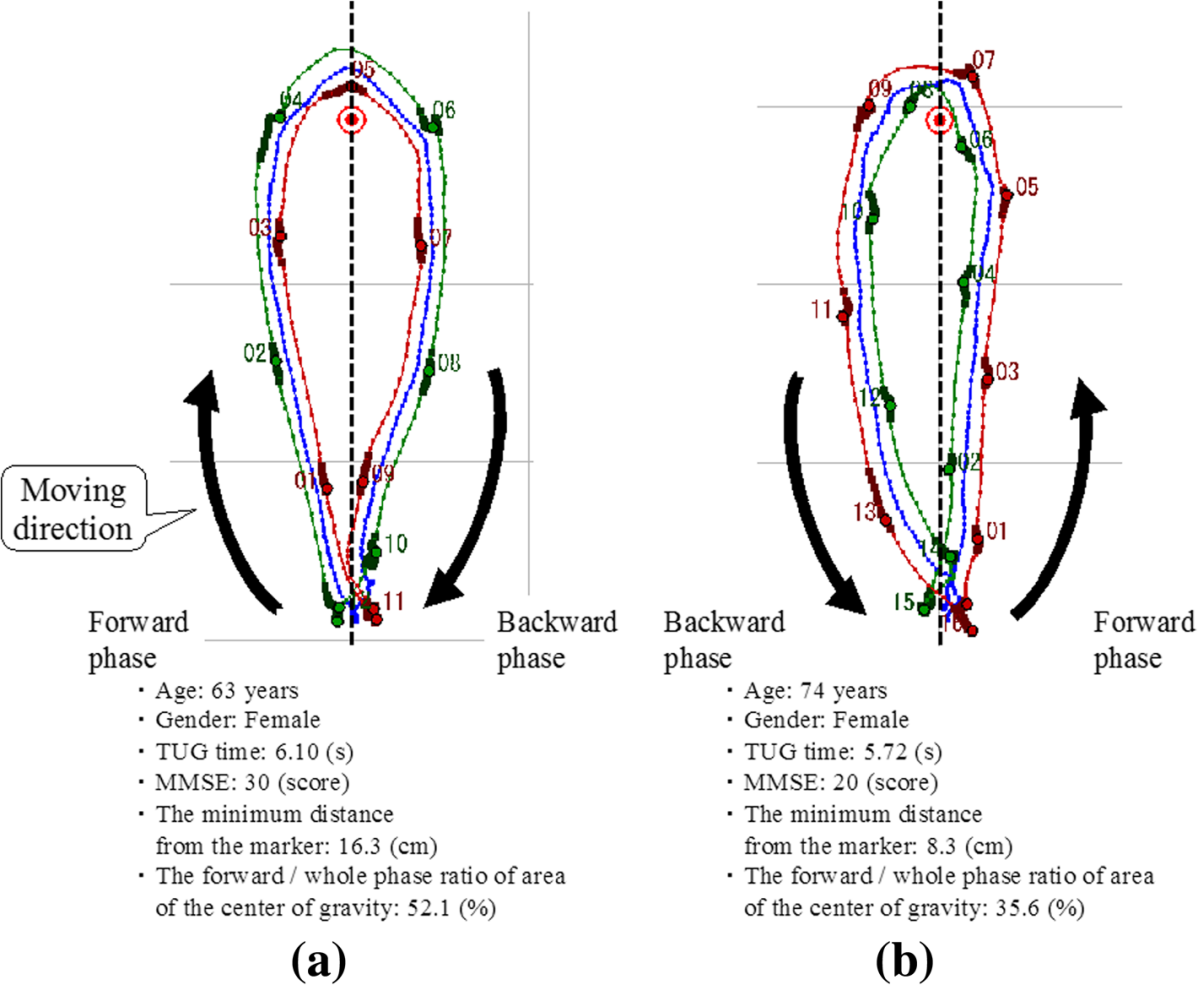
Examples of both leg trajectories showing older adults with normal cognition or mild cognitive impairment. **a** Normal cognitive function, (**b**) Mild cognitive impairment

Typical both leg trajectories during the TUG test of older adults with normal cognition or mild cognitive impairment are shown in Fig. 4; the trajectories of older adults with mild cognitive impairment (right) are relatively asymmetric and closer to the marker.

Furthermore, we conducted multivariate logistic regression analysis adjusted for age, sex, BMI, education, and TUG time. In the analyses, either minimum distance from the marker, the area of the center of gravity in the forward phase, or the forward/whole phase ratio of the area of the center of gravity were used as independent variables. These parameters showed significant differences in the unpaired t-test. The analyses showed that a short minimum distance from the marker (OR: 0.82, 95% CI: 0.69–0.98, *p* = 0.025), narrow area of the center of gravity in the forward phase (OR: 0.01, 95% CI: 0.00–0.36, *p* = 0.012), and large forward/whole phase ratio of the area of the center of gravity (OR: 0.94, 95% CI: 0.88–0.99, *p* = 0.048) were independently associated with mild cognitive impairment (Table 3).

**Table 3:** Multivariate logistic regression models to determine the association between mild cognitive impairment and the trajectory-based spatial parameters during the TUG test

	OR [95% CI]	*p* value		OR [95% CI]	*p* value		OR [95% CI]	*p* value
“The minimum distance from the marker (cm)”	0.82 [0.69–0.98]	0.025*	“The area of the center of gravity in forward phase (m2)”	0.01 [0.00–0.36]	0.012*	“The forward/whole phase ratio of the area of the center of gravity (%)”	0.94 [0.88–0.99]	0.048*
Age	0.93 [0.83–1.04]	0.213	Age	0.95 [0.85–1.05]	0.213	Age	0.97 [0.88–1.07]	0.540
Gender	-	0.522	Gender	-	0.285	Gender	-	0.524
male	1 [Reference]	-	male	1 [Reference]	-	male	1 [Reference]	-
female	0.54 [0.08–3.58]	-	female	0.33 [0.04–2.50]	-	female	0.54 [0.08–3.66]	-
BMI	0.92 [0.77–1.11]	0.396	BMI	0.95 [0.80–1.14]	0.607	BMI	0.95 [0.80–1.13]	0.567
Education	0.98 [0.77–1.24]	0.856	Education	0.99 [0.78–1.29]	0.995	Education	1.03 [0.80–1.33]	0.856
TUG time	1.34 [0.88–2.05]	0.178	TUG time	1.17 [0.76–1.79]	0.475	TUG time	1.18 [0.77–1.79]	0.454

The presence or absence of mild cognitive impairment was used as the dependent variable (normal = 0, mild cognitive impairment = 1)

*OR* Odds ratio, *95% CI* 95% confidence interval, *BMI* = body mass index, TUG = Time Up and Go test

*p < 0.05, **p < 0.01

## Discussion

The results of this study show that some of trajectory-based spatial parameters measured by LRS during the TUG test were independently associated with cognitive impairment in older adults. A short minimum distance from the marker and asymmetrical trajectories (narrow area of the center of gravity from *x*-axis in the forward phase) were independently associated with mild cognitive impairment. To our knowledge, this is the first study to quantify these parameters during the TUG test. These findings suggest that older adults with cognitive impairment have poor TUG performance.

Recent studies have shown that TUG performance is associated with cognitive function, especially executive function, quantitatively [7–9]. As TUG is composed of multiple movement components, such as standing, walking, turning, and sitting, the involvement of various cognitive domains is reasonable, given the complexity of the task. Previous studies have shown that visually-encoded working memory (as in a spatial task) is associated with TUG performance in terms of behavioral [20] and functional brain activation data [21]. In a functional magnetic resonance imaging study, TUG performance was correlated with prefrontal brain activation, though simple walking performance was not [21]. This indicates that the relationship with working memory is stronger in the functional mobility task, which requires more complex motor control, than in the simple walking task.

Our results showed that shorter minimum distances from the marker and asymmetrical trajectories were independently associated with mild cognitive impairment. It has been indicated that spatial parameters change during walking, depending on the subject’s cognitive function. In previous studies with various devices, step-width variability measured by a motion capture system [22], stride time variability measured by an electronic walkway mat [23], gait symmetry and regularity measured by a tri-axis accelerometer [14], and various spatial-temporal parameters measured by an inertial sensor [11] were all related to cognitive performance. Poor spatial gait parameters accompanied by cognitive dysfunction may influence trajectory during walking. In particular, poor executive dysfunction may cause difficulties in planning the tasks of TUG, such as turning, resulting in the short minimum distance from the marker and the asymmetric trajectories. While we often see asymmetrical TUG tests in older adults with cognitive impairment in the clinical setting, it remains to be investigated quantitatively. In this study, by using the LRS leg tracking system during the TUG test, we measured trajectory-based spatial parameters and examined their relationships with cognitive impairment. However, we cannot fully elucidate these relationships because we did not measure executive function, and the number of participants in this study was relatively small. Thus, future studies are needed to investigate these associations in detail.

This is the first study to easily quantify trajectory-based spatial parameters during the TUG test by using an LRS. Our new gait analysis system was able to detect aspects of spatial gait that clinicians would not be able to evaluate just using a stopwatch. In addition, in the LRS system, the LRS position is able to be calibrated using two calibration poles in the TUG field coordinate system easily. Therefore, the system is able to directory measures trajectory-based spatial parameters in the TUG field: distance from the marker, distance from the *x*-axis, and area of region surrounded by the trajectory of the center of gravity and the *x*-axis. To measure these parameters using IMUs (Inertial Measurement Units) [11, 14], it is also necessary to calculate the initial posture of the IMUs in the TUG field coordinate system every time before the test. However, it is difficult to calibrate with high precision when the IMUs attached to the legs of the participants, and it takes time and labor to measure TUG tests several times per participant. On the other hand, compared to the IMU system, the LRS system cannot measure the leg angular parameters directory. In addition, when a long-distance walk test which is longer than TUG test is measured, the stationary LRS measurement system is impossible to measure the walk outside of the sensor range. However, it is necessary to assess many participants in a short time in actual community health centers. The LRS system is a non-contact measurement system and realizes smooth measurement at the scene. In addition, the system can be applied to quantitatively assess abnormal gait in patients with neurological disorders such as Parkinson’s disease and stroke. Further studies are required to understand the neural mechanisms underlying these associations and to evaluate the feasibility of trajectory-based mobility parameter assessments in the context of other disorders.

There were several limitations in the current study. First, the study had a cross-sectional, as opposed to a longitudinal observational, study design. Ultimately, longitudinal measurements are necessary to determine whether changes in spatial parameters during the TUG test precede cognitive decline. The second limitation of this study was the small sample size, which may introduce some error of inference, reduce the power of analysis, and limit generalization. Third, we did not measure detailed aspects of cognition such as executive function. Thus, we cannot explain the reason why the only three parameters reached statistical significance and other parameters do not. In future studies, various cognitive functions should be analyzed in order to assess their relationship to motor functions or other trajectory-based spatial parameters. Finally, the design of this study was not based on population sampling, and the participants in this study did not depend in the sight of their activities of daily life. Thus, future studies should address other frail populations, such as individuals in nursing homes, as our participants were relatively healthy, elderly individuals.

## Conclusions

In conclusion, our results indicate that some of TUG trajectory-based spatial parameters, as measured by LRS, were independently associated with cognitive impairment in older adults. Older adults with cognitive impairment exhibited shorter minimum distances from the marker and asymmetrical trajectories during the TUG test. Further studies are required to understand these associations in detail, including the relevant neural mechanisms.

## Abbreviations

LRS: Laser range sensor
MMSE: Mini-mental state examination
TUG: Timed up and go

## References

[1] Groves WC, Brandt J, Steinberg M, Warren A, Rosenblatt A, Baker A, Lyketsos CG (2000). Vascular dementia and Alzheimer's disease: is there a difference? A comparison of symptoms by disease duration. J Neuropsychiatry Clin Neurosci.

[2] Ferri CP, Prince M, Brayne C, Brodaty H, Fratiglioni L, Ganguli M, Hall K, Hasegawa K, Hendrie H, Huang Y (2005). Global prevalence of dementia: a Delphi consensus study. Lancet.

[3] Brookmeyer R, Johnson E, Ziegler-Graham K, Arrighi HM (2007). Forecasting the global burden of Alzheimer's disease. Alzheimers Dement.

[4] Todd S, Barr S, Roberts M, Passmore AP (2013). Survival in dementia and predictors of mortality: a review. Int J Geriatr Psychiatry.

[5] Podsiadlo D, Richardson S (1991). The timed “up & go”: a test of basic functional mobility for frail elderly persons. J Am Geriatr Soc.

[6] Katsumata Y, Todoriki H, Yasura S, Dodge HH (2011). Timed up and go test predicts cognitive decline in healthy adults aged 80 and older in Okinawa: keys to optimal cognitive aging (KOCOA) project. J Am Geriatr Soc.

[7] Herman T, Giladi N, Hausdorff JM (2011). Properties of the ‘timed up and go’ test: more than meets the eye. Gerontology.

[8] McGough EL, Kelly VE, Logsdon RG, McCurry SM, Cochrane BB, Engel JM, Teri L (2011). Associations between physical performance and executive function in older adults with mild cognitive impairment: gait speed and the timed “up & go” test. Phys Ther.

[9] Donoghue OA, Horgan NF, Savva GM, Cronin H, O'Regan C, Kenny RA (2012). Association between timed up-and-go and memory, executive function, and processing speed. J Am Geriatr Soc.

[10] Eggermont LH, Gavett BE, Volkers KM, Blankevoort CG, Scherder EJ, Jefferson AL, Steinberg E, Nair A, Green RC, Stern RA (2010). Lower-extremity function in cognitively healthy aging, mild cognitive impairment, and Alzheimer’s disease. Arch Phys Med Rehabil.

[11] Greene BR, Kenny RA (2012). Assessment of cognitive decline through quantitative analysis of the timed up and go test. IEEE Trans Biomed Eng.

[12] Van Uem JM, Walgaard S, Ainsworth E, Hasmann SE, Heger T, Nussbaum S, Hobert MA, Mico-Amigo EM, Van Lummel RC, Berg D (2016). Quantitative timed-up-and-go parameters in relation to cognitive parameters and health-related quality of life in mild-to-moderate Parkinson’s disease. PLoS One.

[13] Yorozu A, Moriguchi T, Takahashi M (2015). Improved leg tracking considering gait phase and spline-based interpolation during turning motion in walk tests. Sensors (Basel).

[14] Gillain S, Warzee E, Lekeu F, Wojtasik V, Maquet D, Croisier JL, Salmon E, Petermans J (2009). The value of instrumental gait analysis in elderly healthy, MCI or Alzheimer's disease subjects and a comparison with other clinical tests used in single and dual-task conditions. Ann Phys Rehabil Med.

[15] Yorozu A, Nishiguchi S, Yamada M, Aoyama T, Moriguchi T, Takahashi M (2015). Gait measurement system for the multi-target stepping task using a laser range sensor. Sensors (Basel).

[16] Matsumura T, Moriguchi T, Yamada M, Uemura K, Nishiguchi S, Aoyama T, Takahashi M (2013). Development of measurement system for task oriented step tracking using laser range finder. J Neuroeng Rehabil.

[17] Folstein MF, Folstein SE, McHugh PR (1975). “mini-mental state”. A practical method for grading the cognitive state of patients for the clinician. J Psychiatr Res.

[18] Spering CC, Hobson V, Lucas JA, Menon CV, Hall JR, O'Bryant SE (2012). Diagnostic accuracy of the MMSE in detecting probable and possible Alzheimer's disease in ethnically diverse highly educated individuals: an analysis of the NACC database. J Gerontol A Biol Sci Med Sci.

[19] Yukutake T, Yamada M, Fukutani N, Nishiguchi S, Kayama H, Tanigawa T, Adachi D, Hotta T, Morino S, Tashiro Y (2014). Arterial stiffness determined according to the cardio-ankle vascular index(CAVI) is associated with mild cognitive decline in community-dwelling elderly subjects. J Atheroscler Thromb.

[20] Kawagoe T, Sekiyama K (2014). Visually encoded working memory is closely associated with mobility in older adults. Exp Brain Res.

[21] Kawagoe T, Suzuki M, Nishiguchi S, Abe N, Otsuka Y, Nakai R, Yamada M, Yoshikawa S, Sekiyama K (2015). Brain activation during visual working memory correlates with behavioral mobility performance in older adults. Front Aging Neurosci.

[22] Qu X (2014). Age-related cognitive task effects on gait characteristics: do different working memory components make a difference?. J Neuroeng Rehabil.

[23] Beauchet O, Allali G, Montero-Odasso M, Sejdic E, Fantino B, Annweiler C (2014). Motor phenotype of decline in cognitive performance among community-dwellers without dementia: population-based study and meta-analysis. PLoS One.

